# Rapid and Accurate Genotoxicity Assessment Using the Neutral Comet Assay in *Cyprinus carpio* Cells

**DOI:** 10.3390/life15040603

**Published:** 2025-04-04

**Authors:** Byeonghyeon So, Ji Ho Park, Minseon Kim, Hojun Lee, Jee Hee Yoon, Yoo Jin Lee, Duyeol Kim, Hyung Wook Kwon, Jihae Park, Taejun Han, Yun Haeng Lee, Joon Tae Park

**Affiliations:** 1 Division of Life Sciences, College of Life Sciences and Bioengineering, Incheon National University, Incheon 22012, Republic of Korea; tundra@inu.ac.kr (B.S.); 202428002@inu.ac.kr (J.H.P.); alstjs0323@inu.ac.kr (M.K.); yoojn0905@inu.ac.kr (J.H.Y.); juli9709@inu.ac.kr (Y.J.L.); papaya1130@inu.ac.kr (D.K.); hwkwon@inu.ac.kr (H.W.K.); 2 Bio Environmental Science and Technology (BEST) Lab, Ghent University Global Campus, 119-5, Songdomunhwa-ro, Incheon 21985, Republic of Korea; leehj9311@hanmail.net (H.L.); taejun.han@ghent.ac.kr (T.H.); 3 Convergence Research Center for Insect Vectors, Incheon National University, Incheon 22012, Republic of Korea; 4 Center for Environmental and Energy Research, Ghent University Global Campus, 119-5, Songdomunhwa-ro, Incheon 21985, Republic of Korea; jihae.park@ghent.ac.kr; 5 Department of Animal Sciences and Aquatic Ecology, Ghent University, Coupure Links 653-Block F, B-9000 Gent, Belgium

**Keywords:** genotoxicity assessment, *Cyprinus carpio*, neutral comet assay

## Abstract

Genotoxins cause significant damage to the genetic material of aquatic organisms, requiring rapid and accurate assessment. Fish-derived cells sensitive to genotoxins have proven to be a useful tool for measuring genotoxicity, but the long treatment times required for measurement limit their application in situations requiring rapid testing. Previous studies have shown that fish cells can be kept unstarved for up to 6 h using media containing 1% FBS. In this study, the 1% FBS/6 h parameter was used for genotoxicity assessment. Therefore, genotoxicity assessment was performed after only 6 h of genotoxin treatment in a medium containing 1% FBS. The new genotoxicity assessment method provided faster and more accurate genotoxicity data for climbazole and metolachlor than the existing assessment system using the 15% FBS/96 h parameter. Furthermore, these advantages of the new platform enabled the determination of the genotoxicity of various genotoxins, such as dibenz[a,h]anthracene and ethoprophos. In summary, we have developed a genotoxicity assessment that can generate genotoxicity data rapidly and accurately. This new platform will serve as a foundation for rapid genotoxicity assessment of many genotoxins.

## 1. Introduction

Genotoxins have significant effects on a variety of aquatic plants and animals [1]. Continuous exposure to genotoxins leads to a steady increase in the levels of these toxins in the bodies of aquatic organisms. Genetic material is easily damaged by genotoxins that are retained in the body. This damage can cause morphological and spinal deformities, leading to mortality [2]. In addition to the harmful effects on aquatic organisms, genotoxins can also be toxic to humans who ingest them. Consumption of aquatic organisms contaminated with genotoxins can cause a number of health problems, including infertility, cancer, organ damage, and abnormal development [3]. Effective genotoxin monitoring techniques need to be developed to protect aquatic organisms and humans.

Fish are physiologically sensitive to genotoxic substances and respond quickly, allowing them to detect potential risks from genotoxic substances [4,5]. Although a key tool for improving water quality, fish-based genotoxicity assessments have their drawbacks. Fish respond to a variety of stimuli, in addition to genotoxic substances. Their diverse responses can make data evaluation difficult and lead to inaccurate measurement results [6]. Considering these variables, using fish-based genotoxicity assessments alone may not be accurate. Therefore, more efficient methods that can be applied alone or in combination with fish-based genotoxicity assessments are needed. Fish-derived cell lines have been used as fish surrogates in genotoxicity assessments. Fish-derived cells provide more reliable test conditions by eliminating the variance caused by various actions and reactions [7]. For example, RTG-2 fish cell lines generated from rainbow trout have been used to assess several genotoxins [8,9,10]. Additionally, the genotoxicity of benzo[a]pyrene (B[a]P) and ethyl methanesulfonate was first demonstrated in the Poeciliopsis lucida-derived cell line PLHC-1 [11].

The comet assay is a sensitive technique for detecting DNA damage at the individual cell level [12]. It can be used to screen for genotoxicity in all cell types. It has been widely used in genotoxicology and cancer research to assess DNA strand breaks. Depending on the target, the comet assay using fish cells can vary from 1 to 36 days [7]. However, the number of genotoxins that can be tested in a given time is limited by the long duration of the comet assay. In addition, current genotoxicity assessments cannot meet the needs of rapid assessment. There is an urgent need to develop a new platform that enables rapid testing.

*Cyprinus carpio* (*C. carpio*) is a freshwater fish that is found worldwide, and is especially widely distributed in East Asia, Europe, and North America [13]. This fish, which lives in clear rivers and participates in ecological interactions, functions as an essential member of river ecosystems [14]. Furthermore, *C. carpio* acts as an important indicator species for determining ecotoxicity because it responds rapidly to negative consequences due to water pollution. In a recent study, a new ecotoxicity assessment method using *C. carpio* cells was developed [15]. Based on the finding that *C. carpio* cells were not adversely affected by starvation for up to 6 h in a medium containing 1% FBS, this parameter was applied to the ecotoxicological test [15]. Because the growth-promoting effects of nutrients present in the medium were minimized to the extent that they did not interfere with cellular responses to toxicants, rapid and accurate ecotoxicity testing was possible [15]. Applying these two parameters to genotoxicity assessment will enable the development of rapid measurement methods.

In this study, we developed a platform for assessing genotoxicity using *C. carpio* cells. Compared with traditional genotoxicity assessments, this platform enables rapid and accurate genotoxicity assessments. Here, a novel assessment tool that enables rapid genotoxicity assessments for various genotoxins is proposed.

## 2. Materials and Methods

### 2.1. Cells Cultured from C. carpio

*C. carpio (Cyprinus carpio)* cells used in a previous study were utilized [15]. In accordance with the protocols of the previous study, *C. carpio (Cyprinus carpio)* cells were cultivated [15]. Cell numbers were measured using the Cedex HiRes Analyzer (05650216001; Roche, Basel, Switzerland). All experiments using *C. carpio* cells were approved by the Incheon National University Animal Experiment Ethics Committee (Protocol number: 2024-01-1431, 5 January 2024).

### 2.2. Ecotoxicological Assessment

Ecotoxicological evaluations were performed using cells cultured in 96-well plates for 6 h, in a medium supplemented with 1% FBS, at a density of 2000 cells per well, according to the protocol proposed in our previous study [15]. Afterwards, cells were exposed to the toxicant at concentrations of 0, 62.5, 125, 250, 500, and 1000 ppm. Climbazole (36127; Sigma, St. Louis, MO, USA), metolachlor (36163; Sigma, St. Louis, MO, USA), dibenz[a,c]anthracene (D31206; Sigma, St. Louis, MO, USA), and ethoprophos (45306; Sigma, St. Louis, MO, USA)

### 2.3. Neutral Comet Assay

In media supplemented with 15% or 1% FBS, *C. carpio* cells were seeded at a density of 67,000 cells per well in a 6-well plate. The cells were then exposed to toxicants for 96 h or 6 h. A dimethyl sulfoxide (DMSO) control was used by diluting DMSO (D8418; Sigma, St. Louis, MO, USA) in the medium to a concentration of 0.01%. The CometAssay Single Cell Gel Electrophoresis Assay Kit (4250–050–K; R&D systems, Minneapolis, MN, USA) was utilized. The image j program (National Institute of Health, Bethesda, MD, USA) was used to quantify the DNA comet lengths in pixel units.

### 2.4. Morphology Analysis of Cells

To observe morphological changes, cells were seeded at a density of 2000 cells per well in a 96-well plate. Cells were exposed to toxicants at concentrations of 0, 62.5, 125, 250, 500, and 1000 ppm for 6 h. Then, cells were observed under a microscope.

### 2.5. Statistical Analyses

Student’s *t*-test was calculated using a standard statistical software package (GraphPad Prism 9; San Diego, CA, USA). Student’s *t*-test assumes that the sample population is normally distributed and has homogeneous variances. The no observed effect concentration (NOEC), effective concentration to induce 10% maximal response (EC_10_), semi-effective concentration (EC_50_), and 95% confidence intervals (CI) were calculated using a standard statistical software package (GraphPad Prism 9). To determine the NOEC, EC_10_, EC_50_, and 95% CI, all data were collected in biological triplicates using three samples in each experiment.

## 3. Results

### 3.1. Diagrammatic Representation of Genotoxic Assessment of Traditional and Novel Techniques

**Figure 1:**
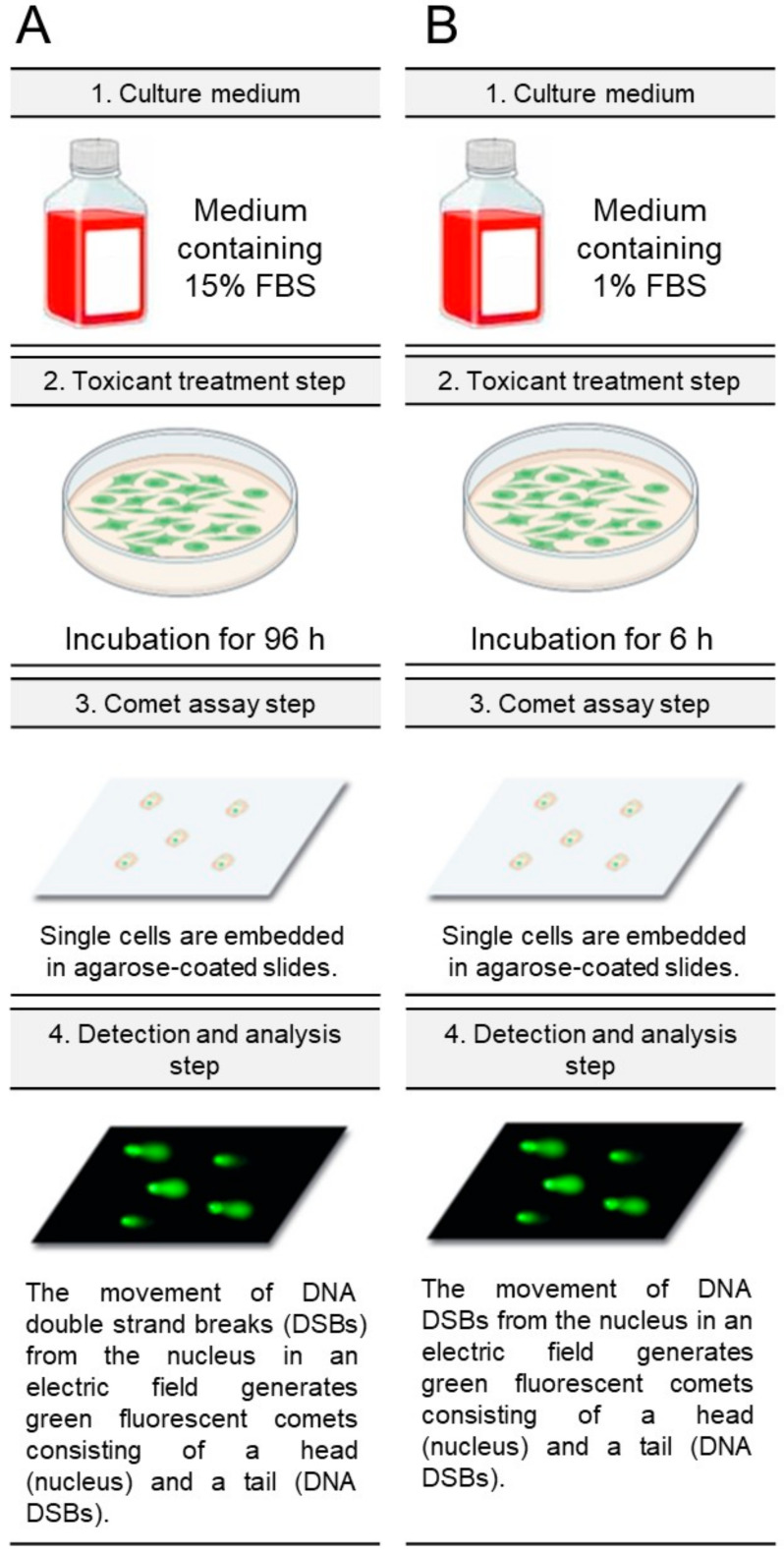
Diagrammatic representation of genotoxic assessment of traditional and novel techniques. (**A**) Using medium containing 15% FBS, *C. carpio* cells were exposed to the toxicant for 96 h. DNA double strand breaks (DSBs) were measured using neutral comet assay. Specifically, the movement of DNA DSBs from the nucleus in an electric field generates green fluorescent comets consisting of a head (nucleus) and a tail (DNA DSBs). (**B**) Using medium containing 1% FBS, *C. carpio* cells were exposed to the toxicant for 6 h. DNA DSBs were measured using neutral comet assay. Specifically, the movement of DNA DSBs from the nucleus in an electric field generates green fluorescent comets consisting of a head (nucleus) and a tail (DNA DSBs).

In our previous study, we established a new ecotoxicological assessment using *C. carpio* cells under 1% FBS/6 h conditions [15]. The improved conditions, which reduced the growth-promoting effects of excess nutrients in the medium, were the underlying mechanism of the improvement [15]. The new ecotoxicological assessment provided rapid and accurate data compared to the conventional assessment. Therefore, we decided to apply these parameters to the genotoxicity assessment. As a control, the 15% FBS/96 h parameter (medium containing 15% FBS and exposure to the toxicant for 96 h) was selected, because 15% FBS is the commonly used concentration in fish cell culture [16,17], and 96 h is within the genotoxicity assessment period of 1–36 days [7] (Figure 1A and Table 1). As an experimental group, the 1% FBS/6 h parameter (medium containing 1% FBS and exposure to the toxicant for 6 h) was used (Figure 1B). Then, in order to identify DNA double-strand breaks (DSBs) at the individual cell level, a neutral comet assay was performed (Figure 1A,B).

### 3.2. Comparing Traditional and New Techniques for Climbazole’s Genotoxicity in C. carpio Cells

**Figure 2:**
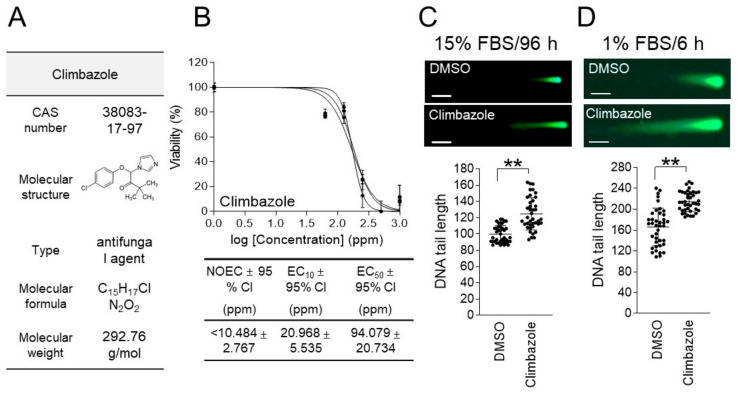
Comparing traditional and new techniques for assessment of climbazole’s genotoxicity in *C. carpio* cells. (**A**) Climbazole’s CAS numbers, molecular weights, types, molecular formulas, and molecular structures are shown. (**B**) After treating *C. carpio* cells with varying concentrations of climbazole (0, 62.5, 125, 250, 500, and 1000 ppm) for 6 h, cell viability was evaluated using a medium containing 1% FBS. GraphPad Prism 9.0 was used to compute the NOEC, EC_10_, EC_50_, and 95% CI. Three samples were used in each experiment, and all data are given in biological triplicate. (**C**) Using a medium containing 15% FBS, *C. carpio* cells were treated with climbazole for 96 h at half the EC_50_ concentration. A neutral comet assay was then carried out. Green fluorescent comets consist of a head (nucleus) and a tail (DNA DSB). Student’s *t*-test, ** *p* < 0.01. *n* = 40, mean ± S.D. (**D**) Using a medium containing 1% FBS, *C. carpio* cells were treated with climbazole for 6 h at half the EC_50_ concentration. A neutral comet assay was then carried out. Green fluorescent comets consist of a head (nucleus) and a tail (DNA DSB). Student’s *t*-test, ** *p* < 0.01. *n* = 40, mean ± S.D.

Climbazole (CAS number: 38083-17-9; molecular weight: 292.76 g/mol) is a widely used antifungal agent that acts by inhibiting sterol biosynthetic enzymes and disrupting the structure and function of the fungal cell membrane [23] (Figure 2A). Climbazole, a common ingredient in shampoos, is an imidazole antifungal agent with antidandruff properties [24]. Therefore, climbazole is known to be one of the causes of aquatic pollution through discharge into wastewater.

The semi-effective concentration (EC_50_) is the concentration of a toxicant that induces a biological response at the midpoint between the baseline and the maximum. It is used as a criterion for selecting the concentration of a genotoxic substance in a genotoxicity test [25,26]. In a genotoxicity evaluation, depending on the purpose, half the EC_50_ concentration, the EC_50_ concentration, or twice the EC_50_ concentration is used [25,26]. To determine the concentration of climbazole to be used in the genotoxicity evaluation, an ecotoxicological evaluation was performed using the 1% FBS/6 h parameter established in our previous study [15].

*C. carpio* cells were treated with various doses of climbazole (0, 62.5, 125, 250, 500, and 1000 ppm) for 6 h in a medium containing 1% FBS. The effect of climbazole on cell viability followed a sigmoid pattern, with an EC_50_ value of 94.079 ± 20.734 (mean ± 95% CI) ppm (Figure 2B and Table S1). The no observed effective concentration (NOEC) and effective concentration to induce 10% maximal responses (EC_10_) of climbazole were less than 10.484 ± 2.767 (mean ± 95% CI) ppm and 20.968 ± 5.535 (mean ± 95% CI) ppm, respectively (Figure 2B).

To evaluate the genotoxicity of climbazole, a neutral comet assay was performed using 47.0395 ppm, which is half the concentration of the EC_50_ value. In the genotoxicity evaluation using the 15% FBS/96 h parameter, the comet tail length in the cells treated with climbazole was shown to increase by 24.97% compared to the DMSO control (Figure 2C). However, in the genotoxicity evaluation using the 1% FBS/6 h parameter, the comet tail length in the cells treated with climbazole was shown to increase by 29.03% compared to the DMSO control (Figure 2D). These data indicate that the comet assay using the 1% FBS/6 h parameter is more responsive to the genotoxicity of climbazole than the method using the 15% FBS/96 h parameter.

### 3.3. Comparing Traditional and New Techniques for Metolachlor’s Genotoxicity in C. carpio Cells

**Figure 3:**
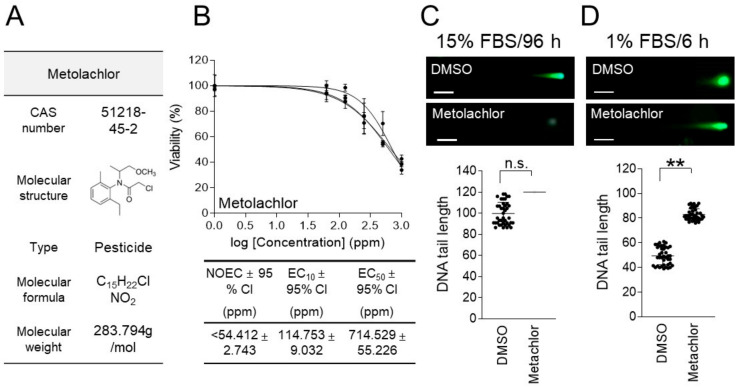
Comparing traditional and new techniques for metolachlor’s genotoxicity in *C. carpio* cells. (**A**) Metolachlor’s CAS numbers, molecular weights, types, molecular formulas, and molecular structures are shown. (**B**) After treating *C. carpio* cells with varying concentrations of metolachlor (0, 62.5, 125, 250, 500, and 1000 ppm) for 6 h, cell viability was evaluated using a medium containing 1% FBS. GraphPad Prism 9.0 was used to compute the NOEC, EC_10_, EC_50_, and 95% CI. Three samples were used in each experiment, and all data are given in biological triplicate. (**C**) Using a medium containing 15% FBS, *C. carpio* cells were treated with metolachlor for 96 h at half the EC_50_ concentration. A neutral comet assay was then carried out. Green fluorescent comets consist of a head (nucleus) and a tail (DNA DSB). Student’s *t*-test, n.s. (not significant). *n* = 40, mean ± S.D. (**D**) Using a medium containing 1% FBS, *C. carpio* cells were treated with metolachlor for 6 h at half the EC_50_ concentration. A neutral comet assay was then carried out. Green fluorescent comets consist of a head (nucleus) and a tail (DNA DSB). Student’s *t*-test, ** *p* < 0.01. *n* = 40, mean ± S.D.

Metolachlor (CAS number 51218-45-2; molecular weight 283.794 g/mol) is a widely used pesticide in agriculture, known for its ability to suppress weed germination and growth by inhibiting long-chain fatty acid synthesis [27] (Figure 3A). Since pesticides are known to ultimately enter aquatic ecosystems and cause long-term problems (including DNA abnormalities and mutations) in aquatic organisms [28], the genotoxicity of metolachlor was evaluated.

An ecotoxicological evaluation was performed to determine the concentration of metolachlor to be used for the genotoxicity assessment, using the 1% FBS/6 h parameter. *C. carpio* cells were treated with various doses of metolachlor (0, 62.5, 125, 250, 500, and 1000 ppm) for 6 h in a medium containing 1% FBS. The effect of metolachlor on cell viability followed a sigmoid pattern, with an EC_50_ value of 714.529 ± 55.226 (mean ± 95% CI) ppm (Figure 3B and Table S2). The NOEC and EC_10_ of metolachlor were less than 54.412 ± 2.743 (mean ± 95% CI) ppm and 114.753 ± 9.032 (mean ± 95% CI) ppm, respectively (Figure 3B).

To evaluate the genotoxicity of metolachlor, a neutral comet assay was performed using 357.265 ppm, which is half the concentration of the EC_50_ value. In the genotoxicity assessment using the 15% FBS/96 h parameter, comet tails were not observed in the cells treated with climbazole, because most of the cells underwent cell death (Figure 3C). However, in the genotoxicity assessment using the 1% FBS/6 h parameter, the comet tail length in the cells treated with metolachlor was found to be increased by 68.26% compared to the DMSO control (Figure 3D). These data indicate that the comet assay using the 1% FBS/6 h parameter provides a more suitable environment for preventing cell death induced by metolachlor genotoxicity than the method using the 15% FBS/96 h parameter.

Based on the genotoxicity results for climbazole and metolachlor, the 1% FBS/6 h parameter was selected for the genotoxicity evaluation.

### 3.4. Genotoxicity Assessment of Dibenz[a,h]Anthracene Using New Method

**Figure 4:**
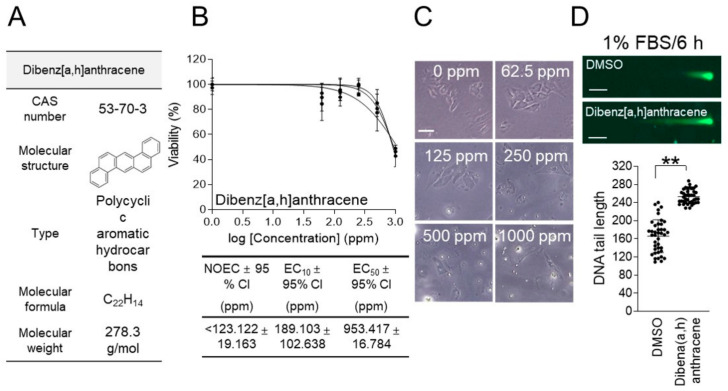
Genotoxicity assessment of dibenz[a,h]anthracene using the new method. (**A**) Dibenz[a,h]anthracene’s CAS numbers, molecular weights, types, molecular formulas, and molecular structures are shown. (**B**) After treating *C. carpio* cells with varying concentrations of dibenz[a,h]anthracene (0, 62.5, 125, 250, 500, and 1000 ppm) for 6 h, cell viability was evaluated using a medium containing 1% FBS. GraphPad Prism 9.0 was used to compute the NOEC, EC_10_, EC_50_, and 95% CI. Three samples were used in each experiment, and all data are given in biological triplicate. (**C**) After treating *C. carpio* cells with varying concentrations of dibenz[a,h]anthracene (0, 62.5, 125, 250, 500, and 1000 ppm) for 6 h, the morphology of *C. carpio* cells was evaluated. Representative images corresponding to each concentration are shown, with a scale bar set at 10 μm. (**D**) Using a medium containing 1% FBS, *C. carpio* cells were treated with dibenz[a,h]anthracene for 6 h at half the EC_50_ concentration. A neutral comet assay was then carried out. Green fluorescent comets consist of a head (nucleus) and a tail (DNA DSB). Student’s *t*-test, ** *p* < 0.01. *n* = 40, mean ± S.D.

The development of new genotoxicity assessment methods led to the measurement of the genotoxicity of chemicals that have not previously been tested for genotoxicity. Dibenzo[a,h]anthracene (CAS number: 53-70-3; molecular weight: 278.3 g/mol) is a polycyclic aromatic hydrocarbon, a toxic compound mainly formed during the combustion of fossil fuels [29] (Figure 4A). The high carcinogenicity and toxicity of this compound pose a serious threat to aquatic ecosystems [29].

An ecotoxicological evaluation was performed to determine the concentration of dibenz[a,h]anthracene to be used for genotoxicity assessment, using the 1% FBS/6 h parameter. *C. carpio* cells were treated with various doses of dibenz[a,h]anthracene (0, 62.5, 125, 250, 500, and 1000 ppm) for 6 h in 1% FBS-containing medium. The effect of dibenz[a,h]anthracene on cell viability followed a sigmoid pattern, with an EC_50_ value of 953.417 ± 16.784 (mean ± 95% CI) ppm (Figure 4B and Table S3). The NOEC and EC_10_ of dibenz[a,h]anthracene were less than 123.122 ± 19.163 (mean ± 95% CI) ppm and 189.103 ± 102.638 (mean ± 95% CI) ppm, respectively (Figure 4B).

Morphological changes induced by dibenzo[a,h]anthracene were not clearly observed at a relatively low concentration of 62.5 ppm, but some morphological changes, such as swelling, were observed at 125 ppm, supporting the NOEC value of 123.122 ppm (Figure 4C). Noticeable cell swelling was observed from 250 ppm, and some cells were observed to rupture at 1000 ppm, supporting the EC_50_ value of 953.417 ppm (Figure 4C).

To evaluate the genotoxicity of dibenz[a,h]anthracene, a neutral comet assay was performed using 476.709 ppm, which is half the concentration of the EC_50_ value. In a genotoxicity assessment using the 1% FBS/6 h parameter, the comet tail length of cells treated with dibenzo[a,h]anthracene was found to increase by 52.31% compared to the DMSO control group, suggesting the genotoxicity of dibenz[a,h]anthracene (Figure 4D).

### 3.5. Genotoxicity Assessment of Ethoprophos Using New Method

**Figure 5:**
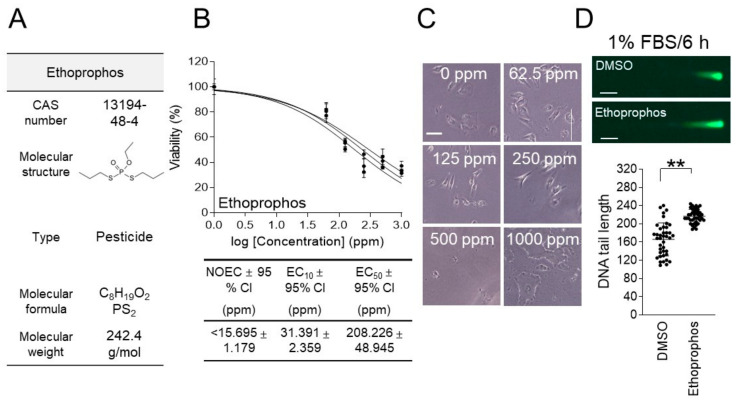
Genotoxicity assessment of ethoprophos using the new method. (**A**) Ethoprophos’s CAS numbers, molecular weights, types, molecular formulas, and molecular structures are shown. (**B**) After treating *C. carpio* cells with varying concentrations of ethoprophos (0, 62.5, 125, 250, 500, and 1000 ppm) for 6 h, cell viability was evaluated using a medium containing 1% FBS. GraphPad Prism 9.0 was used to compute the NOEC, EC_10_, EC_50_, and 95% CI. Three samples were used in each experiment, and all data are given in biological triplicate. (**C**) After treating *C. carpio* cells with varying concentrations of ethoprophos (0, 62.5, 125, 250, 500, and 1000 ppm) for 6 h, the morphology of *C. carpio* cells was evaluated. Representative images corresponding to each concentration are shown, with a scale bar set at 10 μm. (**D**) Using a medium containing 1% FBS, *C. carpio* cells were treated with ethoprophos for 6 h at half the EC_50_ concentration. A neutral comet assay was then carried out. Green fluorescent comets consist of a head (nucleus) and a tail (DNA DSB). Student’s *t*-test, ** *p* < 0.01. *n* = 40, mean ± S.D.

Ethoprophos (CAS number 13194-48-4; molecular weight 242.4 g/mol) is a widely used organophosphate insecticide that is effective in suppressing pest activity by targeting the nervous system of soil-dwelling pests, but its genotoxicity has not been evaluated [30] (Figure 5A).

An ecotoxicological evaluation was performed to determine the concentration of ethoprophos to be used for the genotoxicity assessment, using the 1% FBS/6 h parameter. *C. carpio* cells were treated with various doses of ethoprophos (0, 62.5, 125, 250, 500, and 1000 ppm) for 6 h in a 1% FBS-containing medium. The effect of ethoprophos on cell viability followed a sigmoid pattern, with an EC_50_ value of 208.226 ± 48.945 (mean ± 95% CI) ppm (Figure 5B and Table S4). The NOEC and EC_10_ of dibenz[a,h]anthracene were less than 15.695 ± 1.179 (mean ± 95% CI) ppm and 31.391 ± 2.359 (mean ± 95% CI) ppm, respectively (Figure 5B).

Morphological changes induced by ethoprophos were not clearly observed at relatively low concentrations of 62.5 and 125 ppm (Figure 5C). However, from 250 ppm, cells appeared narrow and atrophied, and at 500 ppm, cells could no longer be clearly observed, which supports the EC_50_ value of 208.226 ppm (Figure 5C).

To evaluate the genotoxicity of ethoprophos, a neutral comet assay was performed using 104.113 ppm, which is half the concentration of the EC_50_ value. In a genotoxicity assessment using the 1% FBS/6 h parameter, the comet tail length of cells treated with ethoprophos was found to increase by 30.29% compared to the DMSO control group, suggesting the genotoxicity of ethoprophos (Figure 5D).

## 4. Discussion

River ecosystems are threatened by genotoxins released from farms and factories near the rivers [31]. Fish are sentinel organisms that detect genotoxins and are important in maintaining the ecological balance of the environment [31]. Fish are sensitive to genotoxins and exhibit distinct physiological responses to them [4,5]. Fish-based genotoxicity tests are a valuable method for assessing genotoxins and provide important perspectives for protecting the ecosystem. Despite their usefulness, fish-based genotoxicity tests have limitations. They are ethically problematic, because live fish must be sacrificed for each test [32]. In addition, genotoxicity assessments may not be accurate, because fish respond inconsistently to various factors [6]. To overcome these limitations, fish cell-based genotoxicity tests have been adopted. Fish cells enable a constant testing environment that is free from variability due to individual fish behavior [33]. In addition, fish cells are more sensitive to genotoxicity and respond to lower genotoxic doses [34]. As a result, fish-based genotoxicity tests are being replaced by fish cell-based genotoxicity tests [7]. However, there were areas that needed improvement in the fish cell genotoxicity assessment method. This assessment method is not suitable for situations that require rapid genotoxicity assessment, because it requires toxicity treatment for 1 to 36 days [7]. Changing the settings to increase the speed of the genotoxicity assessment requires adjusting many variables, and the 1% FBS/6 h parameter established in a previous study was applied to the genotoxicity assessment [15]. *C. carpio* cells responded effectively to the genotoxin under the newly established settings. For example, in the group treated with climbazole, the 1% FBS/6 h parameter increased the average comet tail length more than the 15% FBS/96 h parameter, indicating that the 1% FBS/6 h parameter responded more effectively to the genotoxic effect of the genotoxin. In addition, in the group treated with metolachlor, the 1% FBS/6 h parameter solved the problem of the 15% FBS/96 h parameter, where cells were completely killed and comet tails could not be observed. We propose that this novel method is suitable for situations where rapid assessment is required. However, we acknowledge that more research is needed, as only 1% or 15% FBS concentrations were used to generate the results. It is expected that using other FBS concentrations would allow for faster and more sensitive testing conditions for genotoxicity.

There are approximately 350,000 chemical compounds registered for commercial manufacture, and new chemical compounds are being created [35]. Since very few of these chemicals have been evaluated for genotoxicity, there is a need to rapidly investigate the genotoxicity of most untested compounds. Existing genotoxicity assessment methods require long testing times, as fish cells are exposed to genotoxins for 1–36 days. It is considered difficult to meet the demand for genotoxicity assessment using existing methods, so a new assessment approach is needed to meet the demand. The genotoxicity assessment developed in this study can fully meet these requirements. One scientist can assess 15 to 20 genotoxins per day. In addition, the researcher can start the second test cycle before the first test cycle is completed, allowing twice as many genotoxins to be analyzed in a given time frame. As a result, this new genotoxicity assessment method can rapidly collect a wide range of genotoxicity data, suggesting that it can be an important tool for assessing the genotoxicity of untested genotoxins.

Genotoxins, even in small amounts, build up in the food chain and threaten aquatic ecosystems [36]. These include various hazardous genotoxins, such as antifungals, pesticides, and polycyclic aromatic hydrocarbons. Therefore, development of a universal genotoxicity test is necessary to analyze the genotoxicity of various genotoxins. In this study, we developed a system capable of measuring the genotoxicity of various genotoxins. For example, in the case of climbazole, an antifungal agent used in hygiene products [24], the new method responded better to the genotoxicity of climbazole than the traditional method, as evidenced by the results showing that the new method led to a greater increase in comet tail length than the traditional method. In the case of metolachlor, a widely used pesticide in agriculture [27], it was impossible to measure the comet tail using the traditional method, due to its genotoxic properties that induce cell death, but an increase in the comet tail length was found using the new method. These results are supported by the increase in comet tail length mediated by another pesticide, ethoprophos. Finally, dibenzo[a,h]anthracene, a polycyclic aromatic hydrocarbon produced during the combustion of fossil fuels, has not been measured for genotoxicity before [29]. Our study is the first to detect the genotoxicity of dibenzo[a,h]anthracene, confirming concerns about DNA damage caused by dibenzo[a,h]anthracene. We propose that the new genotoxicity assessment is a universally applicable platform for measuring the genotoxicity of various genotoxins.

Genotoxicity tests are widely used to assess the potential DNA-damaging effects of genotoxins in countries with active water quality monitoring systems. For example, regulatory agencies such as the US Environmental Protection Agency and the European Environment Agency have mandated genotoxicity tests in their water quality assessment programs [37,38]. Specifically, each agency requires that water samples collected at various stages of treatment at water purification plants be tested for the presence of mutagenic, carcinogenic, or DNA-damaging agents. These tests serve as an early warning system to detect genotoxic compounds before the water is used as drinking water. The novel method developed in this study can detect genotoxic substances quickly and accurately, so it can be used to rapidly evaluate genotoxicity in many samples collected during the purification stage. These advantages might be the most important criteria that regulatory agencies in each country consider when choosing a system to evaluate genotoxic substances.

## 5. Conclusions

In conclusion, this study developed an assessment method that can generate genotoxicity data using the 1% FBS/6 h parameter. The platform responded more effectively to genotoxins than existing methods, and was able to measure comet tails which were not possible to measure with existing methods. In addition, this study showed that the platform is suitable for measuring the genotoxicity of various genotoxins. We believe that this new platform can provide a new paradigm for evaluating genotoxins, and can be used as a tool to contribute to environmental monitoring and protection.

## Figures and Tables

**Table 1:** Studies evaluating genotoxicity using the comet assay in fish cell lines.

Cell Line/Species	Genotoxins	Exposure Time	Reference
PAC2/*Danio rerio*	benzo[a]pyrene, ethyl methanesulfonate	6 days	[18]
Erythrocytes/*Danio rerio*	a-tocopherol and anthocyanin	5, 7, 14, or 21 days	[19]
Erythrocytes/*Cyprinus carpio*	karanjin	1, 7, 14, or 21 days	[20]
RTgill-W1/*Oncorhynchus mykiss*	benzothiazole	12 days	[21]
RTL-W1/*Oncorhynchus mykiss*	complex runoff samples	36 days	[22]

## Data Availability

The original contributions presented in the study are included in the article; further inquiries can be directed to the corresponding authors.

## Supplementary Materials

The following supporting information can be downloaded at https://www.mdpi.com/article/10.3390/life15040603/s1: Table S1: Raw data utilized for the ecotoxicological evaluation of climbazole (1% FBS/6 h). Table S2: Raw data utilized for the ecotoxicological evaluation of metolachlor (1% FBS/6 h). Table S3: Raw data utilized for the ecotoxicological evaluation of dibenz[a,h]anthracene (1% FBS/6 h). Table S4: Raw data utilized for the ecotoxicological evaluation of ethoprophos (1% FBS/6 h).
