# Supplementary material for: Rapid and Accurate Genotoxicity Assessment Using the Neutral Comet Assay in Cyprinus carpio Cells
Source: Life (Basel). 2025 Apr 4;15(4):603. doi: 10.3390/life15040603 (PMC12028690; doi:10.3390/life15040603)
Supplement: Supplementary file 1 [file life-15-00603-s001.zip › life-3556688-supplementary.pdf]

Table S1. Raw data utilized for the ecotoxicological evaluation of climbazole (1% FBS/6 h).

Concentration (ppm)	Raw data (Normalized with blank value)								
Biological Repeats	# 1			# 2			# 4		
1000	7833	8753	8851	8767	8733	9016	8263	7744	8695
500	9222	9002	9124	9680	9572	9044	8609	8675	8597
250	8605	7893	8555	9526	8964	8990	8627	8771	8401
125	6434	6398	6676	6428	6542	6159	6616	6610	6211
62.5	3892	3864	4016	4082	4158	4234	4272	3964	4052
0	3798	3574	3828	3978	4036	3752	4024	3882	3992

Table S2. Raw data utilized for the ecotoxicological evaluation of metolachlor (1% FBS/6 h).

Concentration (ppm)	Raw data (Normalized with blank value)								
Biological Repeats	# 1			# 2			# 4		
1000	3156	3216	3272	3286	3296	3354	3446	3338	3398
500	3854	3882	4160	3730	3694	3654	3756	3722	3700
250	4218	3956	3874	4016	3796	4302	3920	3824	4122
125	4124	4052	4146	4054	3866	3918	4014	3978	4040
62.5	4068	4078	4194	3978	4004	4136	4172	3924	4092
0	4118	4120	4158	4302	4052	4308	4412	4182	4108

Table S3. Raw data utilized for the ecotoxicological evaluation of dibenz[a,h]anthracene(1% FBS/6 h).

Concentration (ppm)	Raw data (Normalized with blank value)								
Biological Repeats	# 2			# 3			# 4		
1000	4585	4260	4432	4539	4440	4579	4603	4695	4601
500	4118	4521	4268	4224	4360	4216	4272	4625	4020
250	4274	4174	4302	4306	4168	4396	4300	4260	4254
125	4044	4272	3852	4078	4024	4332	4088	4194	4162
62.5	3894	4138	4166	4274	4038	3852	4286	3758	4148
0	4088	4136	4284	4212	4236	4388	4374	4535	4318

Table S4. Raw data utilized for the ecotoxicological evaluation of ethoprophos (1% FBS/6 h).

Concentration (ppm)	Raw data (Normalized with blank value)								
Biological Repeats	# 1			# 2			# 4		
1000	3142	3020	3006	2968	2928	2986	2894	2866	2812
500	3236	3072	2996	3060	2914	3060	2848	2812	2846
250	3408	3364	3274	3206	3200	3012	2910	2976	3062
125	3198	3060	3072	3080	3090	3138	2918	2852	2862
62.5	3790	3690	3566	3706	3644	3482	3282	3406	3466
0	4110	4090	4182	4210	3994	4010	3860	3868	3860
